# MGMT Promoter Methylation Correlates with an Overall Survival Benefit in Chinese High-Grade Glioblastoma Patients Treated with Radiotherapy and Alkylating Agent-Based Chemotherapy: A Single-Institution Study

**DOI:** 10.1371/journal.pone.0107558

**Published:** 2014-09-11

**Authors:** Dong Shen, Tao Liu, Qingfen Lin, Xiangdong Lu, Qiong Wang, Feng Lin, Weidong Mao

**Affiliations:** Department of Oncology, the Affiliated Jiangyin Hospital of Southeast University Medical College, Jiangyin, P.R. China; H. Lee Moffitt Cancer Center & Research Institute, United States of America

## Abstract

Promoter methylation of the O^6^-methylguanine-DNA-methyltransferase (MGMT) gene has been considered a prognostic marker and has become more important in the treatment of glioblastoma. However, reports on the correlation between MGMT and clinical outcomes in Chinese glioblastoma patients are very scarce. In this study, quantitative methylation data were obtained by the pyrosequencing of tumor tissues from 128 GBM patients. The median overall survival (OS) was 13.1 months, with a 1-year survival of 45.3%. The pyrosequencing data were reproducible based on archived samples yielding data for all glioblastomas. MGMT promoter methylation was detected in 75/128 cases (58.6%), whereas 53/128 (41.4%) cases were unmethylated. Further survival analysis also revealed that methylation was an independent prognostic factor associated with prolonged OS but not with progression-free survival (PFS) (p = 0.029 and p = 0.112, respectively); the hazard radios were 0.63 (95% CI: 0.42–0.96) and 0.72 (95% CI: 0.48–1.09), respectively. These data indicated that MGMT methylation has prognostic significance in patients with newly diagnosed high-grade glioblastoma undergoing alkylating agent-based chemotherapy after surgical resection.

## Introduction

Glioblastoma is considered the highest-mortality cancer of the central nervous system. Although multimodal treatment by surgery, radiotherapy, and chemotherapy is applied, its prognosis is extremely poor [1]. Several reports have shown that epigenetic silencing of MGMT via promoter methylation is associated with improved survival in GBM patients treated with alkylating agents such as temozolomide (TMZ) [2]–[5]. The cytotoxic effects of temozolomide (TMZ) are mediated by DNA methylation at the O^6^ position of guanine as well as by an intact DNA mismatch repair pathway. As the DNA repair protein O^6^-methylguanine-DNA-methyltransferase (MGMT) repairs O^6^-methyl adducts in DNA, MGMT is a critical regulator of the cytotoxic effects of TMZ [5], [6]. Hypermethylation of the MGMT promoter region can silence its expression and result in a deficiency in MGMT-mediated DNA repair and is most frequently detected in high-grade glioma (HGG) and colorectal carcinomas. Hypermethylation of the MGMT promoter in gliomas is associated with sensitivity to alkylating agents including nitrosoureas and TMZ. Reports about the clinical significance of the MGMT promoter methylation status in cohorts of Chinese GBM patients are however very scarce [7]. The objective of this study was to investigate the MGMT promoter methylation status for evaluating the prognostic significance of MGMT in a patient cohort with GBM in a single Chinese institution.

## Patients and methods

### Patients

The study included 128 newly diagnosed, previously untreated, high-grade (grade IV) glioblastoma Han Chinese patients treated from 2008 to 2012 in Department of Oncology, the Affiliated Jiangyin Hospital of Southeast University Medical College. There were 79 males and 49 females. The median age was 56 years (range, 35–71 years). The tumor sizes ranged from 3.7×3.5×2.0 cm to7.2×6.7×5.8 cm. All the patients had undergone prior surgical resection, followed by radiotherapy plus alkylating agent-based chemotherapy. Clinical data were collected retrospectively, and treatment response was monitored with magnetic resonance imaging (MRI) scans after surgery at regular 3-month intervals during follow-up. The progression-free survival (PFS) and overall survival (OS) were calculated from the date of diagnosis.

Approval for the study was obtained from the Medical Ethics Committee of the Affiliated Jiangyin Hospital of Southeast University Medical College. Written informed consent was signed by the patients.

### Pathology and tissues

Tumor samples were collected from the 128 patients. For the tumor tissues, a consultant neuropathologist reconfirmed the diagnosis of glioblastoma WHO grade IV and selected suitable samples for analysis by visual microscopic assessment, with >70% neoplastic cells and <50% necrosis from intraoperative cytology smear preparations or formalin-fixed paraffin-embedded blocks for each case [8]. We aimed to analyze more than one tissue sample for each case, preferably selecting samples from different blocks and/or with different fixation. The characteristics of patients in relation to MGMT promoter methylation are shown in Table 1.

**Table 1:** Summary of glioblastoma patient characteristics.

Characteristics	MGMT promoter methylation(N = 128)(%)		P values
	Methylated(N = 75)	Unmethylated(N = 53)	
**Age**			χ^2^ = 0.586, p = 0.444
<50	29(38.7)	17(32.1)	
> = 50	46(61.3)	36(67.9)	
**Gender**			χ^2^ = 0.714, p = 0.398
Male	42(56.0)	37(69.8)	
Female	33(44.0)	16(30.2)	
**KPS**			χ^2^ = 0.586, p = 0.446
<80	44(58.7)	35(66.0)	
> = 80	31(41.3)	18(34.0)	
**Surgery**			χ^2^ = 0.191, p = 0.662
total resection	20(26.7)	16(30.2)	
subtotal resection	55(73.3)	37(69.8)	

### MGMT promoter methylation Analysis

The QIAamp DNA Mini Kit (Qiagen) was used for genomic DNA isolation from frozen tumor tissues. Spectrophotometry was used for DNA extraction and quantification. Bisulfite modification of 1 mg DNA was performed and each bisulfite modification experiment included universal methylated DNA as positive control and normal brain DNA as negative control. Pyrosequencing was carried out by Gene Tech (Shanghai) Company Limited. The pyrosequencing assay was performed as described by J Dunn et al [2]. The primers used for amplification of bisulphite-treated DNA were forward: 5′-gGGATAGTTGGGATAGTT-3′ (the first g avoids formation of hairpin loops) and reverse: 5′-biotin-ATTTGGTGAGTGTTTGGG-3′ giving a 99-bp amplicon at genomic position 131 155 467–131 155 565. The PCR analysis was performed in duplicate in 25 µl reaction volume. To confirm the correct product before pyrosequencing, 3 ml of PCR products were analyzed on a 2% agarose gel, the remaining 22 ml was subjected to pyrosequencing. The Pyro Q-CpG software 1.0.9 (Biotage) was used to analyze data. Pyrosequencing yielded data for 12 CpG sites within the MGMT promoter. For the data analysis, the percentage methylation obtained for each CpG was averaged across the 12 CpGs in duplicate PCR reactions (average methylation per sample). Compared with the clinical data, the glioblastomas were considered to be methylated if they had at least one sample with an average methylation ≥10% (≥mean±2s.d. for non-neoplastic brain) in more than one independent bisulfite modification [2], [9]–[11]. The average methylation of unmethylated cases was <10% in all samples. The average methylation per case was calculated by averaging the average methylation per sample for the methylated samples for that case. Further, according to extent of methylation, the prognostic stratification was split into 4 groups: fully unmethylated(0% methylation), unmethylated(>0 to <10% methylation), methylated(≥10 to <100% methylation), fully methylated(100% methylation).

### Statistical analysis

The differences in clinicopathologic variables in different groups were evaluated by the Exact Sig (2-sided) χ^2^ test. Kaplan-Meier survival curves were obtained, and differences in PFS or OS were tested for statistical significance using the log-rank test. P<0.05 was considered the statistically significance level. A stepwise Cox regression multivariate analysis for factors significantly associated with survival in the univariate analysis was performed with the parameters of a significance of 0.05 for entry and 0.01 for removal. The data were analyzed using PASW Statistics 18 (Version 18.0.0).

## Results

**Figure 1:**
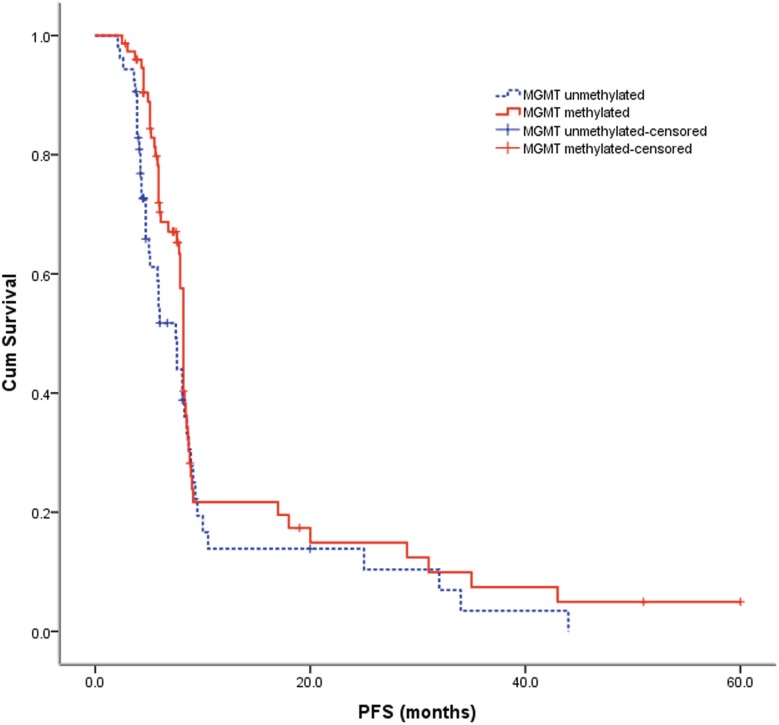
PFS after treatment in patients with methylated and unmethylated MGMT promoter glioblastomas (log-rank P = 0.112).

**Figure 2:**
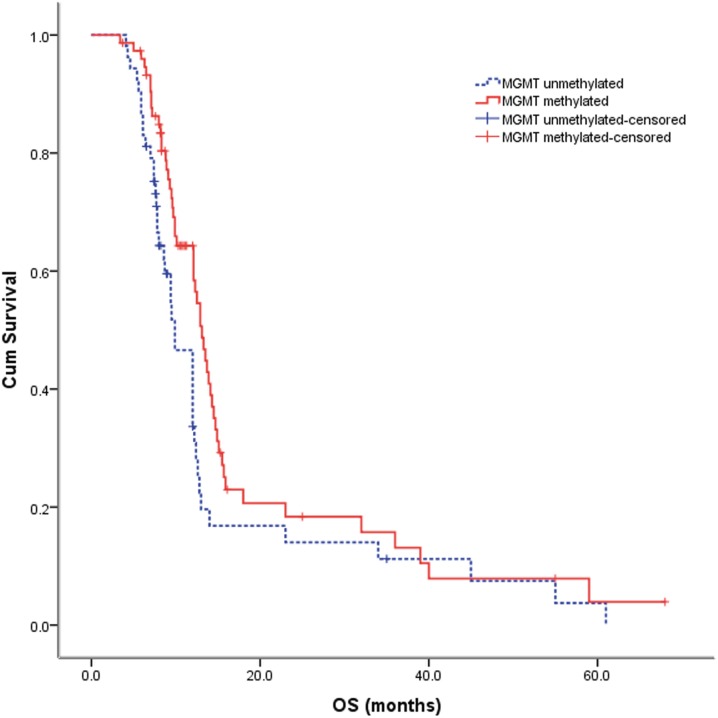
OS after treatment in patients with methylated and unmethylated MGMT promoter glioblastomas (log-rank P = 0.029).

The patient characteristics are summarized in Table 1. 75 out of 128 patients (58.6%) had average methylation across all CpGs in at least one clinical sample greater than10% and were classified as methylated, the average methylation in methylated cases was 44.4±23.4%, and 100% methylation (fully methylated) was detected in 3 patients (3/75, 4.0%). And the other 53 patients (41.4%) was classified as unmethylated, the average methylation in unmethylated cases was 2.1±1.6%, and 0% methylation (fully unmethylated) were detected in 19 patients (19/53, 35.8%). No significant correlation was observed between the MGMT promoter methylation status and any baseline variables, including age at study entry (p = 0.444), gender (p = 0.398), KPS (p = 0.446), and surgery (p = 0.662). Furthermore, Kaplan–Meier analysis and Cox regression showed no significant difference in the progression-free survival between GBM patients with a methylated MGMT promoter and those without a methylated MGMT promoter (p = 0.112, table2, Fig. 1); the hazard radio was 0.72 (95% CI: 0.48–1.09). However, the GBM patients with a methylated MGMT promoter had a better outcome of overall survival at a statistically significant level, with a hazard radio of 0.63 (95% CI: 0.42–0.96) (p = 0.029, table2, Fig. 2). The prognostic stratification according to the extent of methylation was shown in table 3. More information can be seen or calculated in the supporting information file named Data S1.

**Table 2:** MGMT promoter methylation and clinical outcomes in Chinese glioblastoma patients*.

	PFS				OS					
	Median(months)	95% CI	HR	95% CI	Median (months)	95% CI	HR	95% CI	1 year survival(%)	95% CI
Unmethylated(N = 53)	7.5	5.7–9.3	0.72	0.48–1.09	9.9	8.3–11.5	0.63	0.42–0.96	30.2	18.34–44.34
Methylated(N = 75)	8.2	8.0–8.4			13.1	11.7–14.4			45.3	33.79–57.25

*For PFS: LOG RANK χ^2^ = 2.52, p = 0.112; For OS: LOG RANK χ^2^ = 4.79, p = 0.029.

**Table 3:** Extent of MGMT promoter methylation and clinical outcome in Chinese glioblastoma patients*.

	NO.	PFS		OS	
		Median(months)	95% CI	Median(months)	95% CI
Fully unmethylated(0% methylation)	19	4.1	3.8–4.4	6.4	5.3–7.5
Unmethylated(>0 to <10% methylation)	34	7.8	7.3–8.3	10.3	8.8–11.8
Methylated(≥10 to <100% methylation)	72	8.1	7.8–8.4	12.6	12.1–13.5
Fully methylated(100% methylation)	3	51.0	30.2–72.5	59.0	44.1–77.2

*For PFS: LOG RANK χ^2^ = 82.134, p<0.001; For OS: χ^2^ = 23.145, p<0.001.

## Discussion

Various studies have shown that the MGMT promoter methylation status is an independent prognostic factor [3], [5], [12], [13]. It is believed that patients with GBM who have a methylated MGMT promoter benefit from temozolomide, whereas those who do not have a methylated MGMT promoter do not have this benefit. Given the importance to the clinical management of glioblastoma patients, experience in the routine clinic is essential for these advances to have full clinical benefit [7], [14]. This study was performed to define the prognostic and predictive value of MGMT promoter methylation in Chinese glioblastoma patients.

The major strengths of this study include the relatively large sample size, the prospective data collection, the standardized use and pyrosequencing analysis to assess the MGMT promoter methylation status, and the opportunity of dissecting the prognostic and predictive aspects of MGMT promoter methylation as a biomarker. This cohort represents consecutive patients treated in a single center over a 68-month period, and the comparison of survival data supports little selection bias in the study. The cohort with methylation data had a similar median age and range, performance status, and proportion of patients with biopsy vs debulking surgery compared with certain clinical studies [3], [15]–[17]. Progression-free survival was 8.2 months compared with 6.9 months reported by Stupp et al [15], which may reflect the response evaluation and follow-up achieved in routine practice. Overall survival was 13.1 months in contrast to 14.6 months, but this is not an unexpected finding, as outcome in a routine clinical environment is often not better than that in clinical trials. For the entire cohort of 128 patients, MGMT promoter methylation was prognostic for OS but not for PFS (Fig. 1 and Fig. 2).

When considered the 4 prognostic stratification according to extent of methylation, the cases with 100% methylation had the longest survival. Significant differences in PFS were also seen between those with intermediate/high methylation and unmethylated cases, while all stratification had significantly different OS. Therefore, the extent of methylation may have impacts on associations with survival, as other studies showed [2], [18]. However, the observations from another research teams do not lead us to consider promoter methylation of the MGMT gene as a prognostic factor of responsiveness to alkylating agents in GBM [7], [14], [19]. Another report drew a similar conclusion, with no prognostic effect of MGMT promoter methylation being observed in tumors diagnosed in a central pathology review as glioblastoma [2], [14], [20]. To confirm and extend these data in Chinese GBM patients, pyrosequencing, a gold standard for methylation analysis, was employed to detect MGMT promoter methylation in this study. Unlike the methylation-specific PCR (MSP) widely used in many clinical studies, the highly reproducible quantitative pyrosequencing protocol makes it the method of choice for methylation evaluations in many diagnostic and research applications [2], [11]. Our results showed that GBM patients with MGMT promoter methylation had a better outcome with regard to overall survival at a statistically significant level.

MGMT plays an important role in maintaining genomic integrity by removing alkyl adducts from the O^6^ position of deoxy-guanine and preventing the formation of DNA interstrand cross-links and is believed to be the most important factor in the acquisition of clinical resistance to alkylating agents [7], [21]–[22].

The limitation of this study is that the analysis of MGMT protein expression was not performed. In theory, the effect of MGMT promoter methylation on prognosis and chemosensitivity to alkylating agents depends on the expression of the MGMT protein. Therefore, MGMT expression at the protein level may also have similar efficacy for predicting prognosis and chemosensitivity in GBM patients [7], [11], [19]. But the relationship between methylation and protein expression was still questioned [2], [19].

This study also found that the MGMT promoter methylation status shows certain differences when sampling from different parts of tumors. The reason may be related to the presence of pathological heterogeneity and genetic inhomogeneity in different parts of glioblastoma tissues and clinical factors [23]–[25]. Similar results were also reported for malignant melanoma [25]–[27].

Currently, there are few reliable clinical indicators and testing methods for guiding chemotherapy [28], [29]. Random sampling from tissues is used to detect the MGMT gene promoter methylation status to predict whether cancer patients are resistant to alkylating agents [30], [31]. Because tumor heterogeneity exists, such a strategy will inevitably result in false negatives [17], [26], [32]. Therefore, it is necessary to explore detection methods using sampling from multiple sites of the tumor to derive the MGMT gene promoter methylation status to study the corresponding sequential chemotherapy dosage and mode of administration.

## Supporting Information

Data S1 (SAV)
